# Traumatic Extrusive and Lateral Luxation of Maxillary Incisors During Orthodontic Treatment: A Multidisciplinary Management Approach

**DOI:** 10.3390/dj14070415

**Published:** 2026-07-07

**Authors:** Marina Mirzabekian, Narine Arutiunian, Nina Karpoeva, Angelina Petiuleva, Tigran Minasyan, Ekaterina Zernitckaia, Sofiia Khadzhieva

**Affiliations:** 1 Moscow Regional Scientific Research Clinical Institute, MF Vladimirsky, Regional Research Clinical Institute, Moscow 129110, Russia; petyulyova@gmail.com; 2 Department of Orthodontics, Stavropol State Medical University, Stavropol 355017, Russia; aesthetic.dc@mail.ru (N.A.); s.hadjieva@yandex.ru (S.K.); 3 School of Dentistry, Loma Linda University, Loma Linda, CA 92350, USA; nkarpoeva@students.llu.edu (N.K.); tminasyan@students.llu.edu (T.M.); 4 Advanced Education Program in Implant Dentistry, School of Dentistry, Loma Linda University, Loma Linda, CA 92350, USA; ezernitckaia@llu.edu

**Keywords:** tooth luxation, lateral luxation, extrusive luxation, traumatic dental injuries, orthodontic appliances, dental splinting, mouthguards, root canal therapy, dental implants

## Abstract

**Background/Objectives**: Traumatic dental injuries involving the maxillary anterior teeth are common; however, combined extrusive and lateral dislocation during active orthodontic treatment is uncommon and presents unique management challenges. This case report describes the multidisciplinary management of traumatic extrusive and lateral dislocation of the maxillary right lateral incisor (#7) and maxillary right central incisor (#8) in a 27-year-old patient undergoing fixed orthodontic treatment following sports-related trauma. **Methods:** Clinical examination revealed marked coronal and lateral displacement, pathologic mobility, gingival laceration, active bleeding, and deformation of the orthodontic archwire. Both affected teeth had complete root formation. Emergency management included immediate repositioning of the displaced teeth and urgent endodontic treatment. Semi-rigid stabilization was achieved using metallic ligatures and a rectangular stainless-steel orthodontic archwire, supplemented by temporary skeletal anchorage with a mini-screw and posterior bite turbos to reduce occlusal loading. The patient was monitored clinically and radiographically throughout healing and subsequent orthodontic treatment. **Results**: Follow-up examinations demonstrated favorable periodontal healing, stable retention of the traumatized teeth, absence of pathologic mobility, and successful continuation of orthodontic treatment. Radiographic evaluation showed no evidence of inflammatory root resorption or other significant complications. At the 16-month follow-up, the affected teeth remained functional and stable, and implant placement was successfully completed at the congenitally missing maxillary canine site. **Conclusions**: This case highlights the importance of immediate interdisciplinary management of traumatic dental dislocation during orthodontic treatment. Fixed orthodontic appliances, when appropriately incorporated into emergency stabilization protocols, may contribute to successful tooth retention and favorable long-term outcomes.

## 1. Introduction

Traumatic dental injuries (TDIs) involving the maxillary anterior teeth are common clinical emergencies, particularly among young and physically active individuals. Luxation injuries constitute a significant subgroup of TDIs and are frequently associated with disruption of the periodontal ligament, neurovascular supply, and surrounding soft tissues [1]. Extrusive luxation is characterized by partial axial displacement of the tooth out of its socket and is commonly accompanied by increased mobility, elongation of the clinical crown, and gingival bleeding [1,2]. Lateral luxation is defined as displacement of the tooth in a direction other than axial, frequently accompanied by fracture or compression of the alveolar socket wall and damage to the periodontal ligament. When extrusive and lateral components occur together, the injury may involve both coronal displacement out of the socket and displacement toward the labial, palatal, or mesiodistal direction. Such combined injuries may carry an increased risk of pulpal necrosis [3,4], periodontal ligament damage, inflammatory root resorption, and marginal bone complications, particularly in mature permanent teeth [5,6,7,8,9].

Successful management of extrusive and lateral luxations depends on timely repositioning, adequate stabilization, preservation of pulpal and periodontal health, and long-term clinical and radiographic monitoring [1,10]. Delayed or inadequate treatment may increase the risk of pulpal necrosis, inflammatory root resorption, ankylosis, and long-term periodontal complications [1,5,6,7,11,12]. Current International Association of Dental Traumatology (IADT) guidelines recommend immediate repositioning and flexible splinting to optimize periodontal healing and reduce the likelihood of unfavorable outcomes [1,13].

Traumatic injuries occurring during active orthodontic treatment present additional clinical challenges. The presence of fixed orthodontic appliances may complicate emergency management and influence subsequent orthodontic treatment planning and biomechanical control [9,14,15,16]. Despite the growing number of adult patients undergoing orthodontic therapy, reports describing the management of severe extrusive and lateral luxation injuries during active orthodontic treatment remain limited.

This case report describes the multidisciplinary management and 16-month follow-up of combined extrusive and lateral luxation of two maxillary incisors in an adult patient undergoing active fixed orthodontic treatment.

## 2. Case Presentation

This report describes a single clinical case and did not involve a systematic investigation designed to produce generalizable knowledge. Therefore, formal Institutional Review Board approval was not required according to institutional policy. The case was conducted in accordance with the principles of the Declaration of Helsinki. Written informed consent was obtained from the patient for treatment, publication of clinical data, and use of clinical photographs and radiographic images.

A 27-year-old male patient was undergoing comprehensive fixed orthodontic treatment for correction of a complex malocclusion. Initial orthodontic diagnosis revealed an edge-to-edge anterior relationship, mandibular anterior crowding, anterior crossbite on the right side, a Class III molar relationship on the left, a Class II molar relationship on the right, and a congenitally missing maxillary right canine (#6). Significant space loss was noted in the maxillary right canine region secondary to mesial drift of the adjacent teeth following extraction of the retained primary canine (Figure 1). In addition, the patient presented with multiple untreated carious lesions, poor oral hygiene, and noticeable attritional wear of the anterior dentition (Figure 2). At the time of injury, orthodontic treatment was in the early alignment and leveling phase, with one of the principal treatment objectives being creation of adequate mesiodistal space at site #6 for future implant rehabilitation.

During a football training session, the patient sustained a direct traumatic impact to the anterior maxillary region. He presented to the clinic within approximately one hour after the incident, reporting acute pain, inability to achieve normal occlusion, displacement of the maxillary anterior teeth, and gingival bleeding. Clinical examination demonstrated combined extrusive and lateral luxation of the maxillary right lateral incisor (#7) and maxillary right central incisor (#8), characterized by marked coronal displacement, lateral displacement, pronounced clinical instability, gingival laceration, and active bleeding from the surrounding periodontal tissues. Partial deformation and displacement of the orthodontic archwire were also observed as a result of the impact (Figure 3). The involved anterior maxillary segment was tender to palpation.

A periapical radiograph confirmed extrusive and lateral displacement of teeth #7 and #8 without evidence of alveolar bone fracture, root fracture, or retained root fragments (Figure 4).

Given the acute nature of the injury, both luxated teeth were immediately repositioned into their original anatomical positions within the alveolar sockets under local anesthesia [1]. Considering the severity of displacement and the high risk of pulpal necrosis associated with extrusive luxation in mature permanent teeth [1,4,5,8,11], the patient was referred on the same day for urgent endodontic intervention [6]. Immediate root canal therapy of teeth #7 and #8 was subsequently performed by an endodontist as part of the multidisciplinary emergency management in order to minimize the risk of infection-related inflammatory root resorption and long-term pulpal complications [1,3,4,7,9,11,12,17].

The prognosis of the dental pulp following luxation injuries is strongly influenced by root maturity. Immature permanent teeth with open apices possess a greater capacity for pulpal revascularization and may maintain vitality following traumatic displacement. In contrast, mature permanent teeth with closed apices demonstrate a substantially higher risk of pulpal necrosis due to disruption of the neurovascular supply and limited regenerative potential [5,6,8]. In the present case, both affected incisors had complete root formation, and the severity of the injury further increased the risk of pulpal complications [5,6]. Therefore, early endodontic intervention was considered an appropriate component of the multidisciplinary treatment strategy [5,6].

Following repositioning and completion of endodontic therapy, an orthodontic mini-screw temporary anchorage device (TAD) was inserted in the edentulous area corresponding to the missing maxillary right canine (#6) to provide skeletal anchorage while avoiding transmission of additional orthodontic forces to the traumatized anterior teeth (Figure 5) [14,15,18]. Stabilization of the luxated teeth was achieved using metallic ligature wires secured across the maxillary anterior segment in conjunction with a 0.017 × 0.025-inch rectangular stainless-steel orthodontic archwire, which functioned as a semi-rigid splint during periodontal healing (Figure 6) [1,13,19]. To further minimize functional loading on the injured teeth and eliminate occlusal contacts in the anterior segment, posterior bite turbos were bonded on the maxillary first molars to establish temporary posterior disocclusion (Figure 7) [14,20].

## 3. Results

At the 1-month follow-up, the patient reported no subjective complaints. Clinical examination demonstrated stable retention of teeth #7 and #8 within the dental arch, with a marked reduction in mobility from Grade III at baseline to Grade I. Mild tenderness to percussion was still present, and the surrounding gingival tissues exhibited satisfactory healing, although mild residual erythema remained in the affected area. Periodontal probing depths had improved from baseline values exceeding 5 mm to 3.5 mm for tooth #7 and 4.5 mm for tooth #8. A periapical radiograph obtained at this time confirmed correct positioning of the teeth within the alveolar bone, with no radiographic signs of periapical pathology, periodontal ligament space widening, or inflammatory root resorption [12]. Intraoral photographs further demonstrated stable retention of the traumatized teeth and progressive soft tissue healing (Figure 8).

At the 3-month follow-up, the patient remained asymptomatic. Clinical examination demonstrated continued stability of teeth #7 and #8, with complete resolution of the previously observed gingival erythema and no evidence of recurrent displacement or other complications. Further orthodontic treatment was intentionally deferred to allow a total healing period of six months following trauma before reactivation of active orthodontic mechanics [15,16,18,21].

At the 6-month follow-up, the patient remained asymptomatic. Clinical examination demonstrated complete stability of teeth #7 and #8, with no evidence of pathologic mobility (Mobility Grade 0), recurrent displacement, or periodontal compromise. Periodontal probing depths had returned to physiologic values of approximately 3 mm around both teeth, and percussion testing was negative. The surrounding gingival tissues appeared healthy, with normal color, contour, and consistency. Radiographic evaluation revealed no evidence of inflammatory root resorption or other pathologic changes. Given the favorable clinical outcomes, orthodontic treatment was resumed at this stage (Figure 9) [15,16,18].

At the 16-month follow-up after trauma, the patient remained asymptomatic and reported no complaints. Orthodontic treatment had been resumed and progressed uneventfully. Throughout this phase, particular care was taken to apply only light, controlled orthodontic forces to the previously traumatized teeth [14,15,16,18,21,22]. In addition, teeth #7 and #8 remained stabilized with metallic ligature wires during the healing and orthodontic phases to maintain their position within the dental arch [13,19,22]. Sufficient space was established in the region of the missing maxillary right canine (#6) to allow for implant placement, and a dental implant was subsequently placed by an oral surgeon. Clinical examination demonstrated stable tooth position, absence of pathologic mobility (Mobility Grade 0), negative percussion findings, periodontal probing depths maintained at 3 mm, and healthy periodontal tissues without evidence of periodontal breakdown or occlusal dysfunction. Periapical and panoramic radiographs obtained at this stage confirmed proper implant positioning and showed no radiographic evidence of inflammatory root resorption, periapical pathology, or other pathologic changes in the maxillary anterior region (Figure 10a–c).

A detailed summary of the clinical outcomes, including tooth mobility, periodontal probing depths, percussion findings, pulp status, gingival condition throughout the follow-up period, is presented in Table 1.

## 4. Discussion

Extrusive and lateral luxation represents a severe form of traumatic dental injury [1,2], involving partial displacement of the tooth from its socket and disruption of the periodontal ligament and neurovascular supply. Clinically, it is characterized by increased tooth mobility, elongation of the clinical crown, gingival bleeding, and sensitivity to percussion. Radiographically, widening of the periodontal ligament space, particularly at the apical region, is commonly observed. The prognosis of teeth affected by extrusive and lateral luxation depends on multiple factors, including the degree of displacement, root maturity, timing of repositioning, and quality of stabilization [1,5,11].

Root maturity is a major prognostic factor following luxation injuries. Teeth with open apices generally demonstrate a greater potential for pulpal healing and revascularization, whereas mature teeth with closed apices are significantly more susceptible to pulpal necrosis and often require endodontic treatment [5,7,9,11,17]. Consequently, treatment planning and follow-up protocols may differ substantially between immature and mature permanent teeth [5,8,10]. In the present case, the mature status of both incisors supported the decision for early endodontic management.

In orthodontic patients, the management of luxation injuries is particularly complex and may vary among clinicians depending on experience and treatment philosophy [23,24]. Orthodontic forces alter the periodontal ligament environment and may influence the healing response following trauma [15,16,18,21,22]. Moreover, the presence of fixed appliances can complicate emergency repositioning procedures, as displaced brackets and archwires may interfere with proper alignment of the injured teeth [14,15,16]. Conversely, orthodontic appliances may act as a semi-rigid splint, limiting excessive tooth displacement at the time of injury and reducing the risk of complete avulsion [13,19].

In the present case, both maxillary incisors sustained combined extrusive and lateral luxation during a football training session. Notably, despite the severity of the trauma, complete avulsion did not occur. It is possible that the orthodontic archwire and brackets contributed to partial retention of the teeth within the dental arch at the moment of impact. However, this observation should be interpreted cautiously as a causal protective effect cannot be established from a single case report. This observation aligns with findings reported by Leidenz et al., who described the use of orthodontic appliances as part of a stabilization strategy in traumatic dental injuries [19].

Immediate repositioning is a critical determinant of favorable outcomes in extrusive and lateral luxation [1,12]. According to International Association of Dental Traumatology (IADT) guidelines, repositioning should be performed as soon as possible to reduce damage to the periodontal ligament and minimize the risk of external inflammatory root resorption [1]. In the present case, both teeth were repositioned on the same day as the injury, followed by immediate endodontic therapy. Although pulpal vitality may occasionally be preserved in extrusive luxation injuries, the interaction between trauma and orthodontic forces significantly increases the risk of pulpal complications [1,3,5,7,11,12,17,22]. Therefore, early endodontic intervention was deemed appropriate to prevent infection-related resorptive processes [1,5,8,11,12,17].

Stabilization following repositioning represents a critical component in the management of extrusive and lateral luxation injuries. Current guidelines generally recommend the use of a flexible splint for approximately two weeks to allow for optimal periodontal ligament healing [1,13]. In the present case, stabilization was achieved using metallic ligatures secured across the anterior segment in conjunction with a rectangular stainless-steel orthodontic archwire. This approach provided a semi-rigid form of stabilization, while maintaining physiologic mobility of the affected teeth and allowing for periodontal healing without the application of uncontrolled orthodontic forces [13,14,19]. Additionally, a mini-screw was placed at the site of the congenitally missing maxillary right canine (#6) to provide skeletal anchorage and enhance control over tooth positioning. The use of skeletal anchorage in traumatic dental injury management is not routinely described in the literature but may offer potential advantages in orthodontic patients during the healing phase. By providing anchorage independent of the traumatized dentition, temporary anchorage devices may help minimize unwanted tooth movement and reduce the transmission of orthodontic forces to injured teeth while periodontal healing is occurring. In the present case, skeletal anchorage facilitated stabilization of the anterior segment without placing additional mechanical stress on the traumatized incisors. Although the favorable outcome cannot be attributed solely to the use of a mini-screw, this approach may represent a useful adjunctive strategy in selected orthodontic trauma cases and warrants further investigation.

Posterior bite turbos were placed to create disocclusion and eliminate occlusal forces on the traumatized incisors [14,20]. This step is particularly important in patients with edge-to-edge bites or anterior crossbites, where functional contacts could jeopardize healing. Reduction of occlusal loading allows the periodontal ligament to reorganize and reduces the risk of secondary trauma during mastication [20].

At the one-month follow-up, the injured teeth demonstrated marked improvement in stability, with mobility reduced from Grade III to Grade I and favorable radiographic findings. These short-term results suggest that immediate interdisciplinary management can lead to satisfactory early outcomes, even in complex cases involving orthodontic appliances. However, long-term monitoring remains essential, as complications such as replacement resorption, ankylosis, or marginal bone loss may develop months or years after the initial injury [1,12].

Comparison with previously published reports highlights the importance of timing in trauma management. Kadulkar et al. reported successful outcomes in delayed replantation of avulsed teeth, emphasizing the role of meticulous endodontic and periodontal care [25]. In contrast, the present case underscores the benefits of immediate intervention in extrusive luxation, where preservation of the periodontal ligament is still possible.

Recent systematic evidence further highlights the importance of long-term monitoring following severe luxation injuries. Tewari et al. reported that lateral luxation injuries are associated with a substantial risk of pulp necrosis and other post-traumatic complications, particularly in permanent teeth with complete root formation [5,8,26]. Furthermore, recent evidence has demonstrated that traumatic dental injuries may result in a broad spectrum of post-traumatic complications, including pulp canal obliteration, pulp necrosis, inflammatory root resorption, ankylosis, and periodontal breakdown, emphasizing the importance of prolonged follow-up even when initial healing appears favorable [4,5,7]. Consistent with these recommendations, the present case was monitored clinically and radiographically for 16 months and demonstrated stable periodontal and periapical conditions without evidence of significant complications.

Preventive considerations are particularly relevant in orthodontic patients engaged in contact sports [24,27,28]. The presence of fixed appliances increases the risk of both dental and soft tissue injuries. Current expert consensus strongly recommends the use of custom or orthodontic-compatible mouthguards to reduce the incidence and severity of traumatic injuries [27,29]. Despite these recommendations, compliance remains suboptimal. This case highlights the potentially severe consequences of inadequate protective measures and reinforces the need for preventive counseling as part of routine orthodontic care [28].

The use of properly fitted mouthguards is considered the most effective preventive measure for reducing the incidence and severity of sports-related dental injuries [27]. For patients undergoing orthodontic treatment, orthodontic-compatible mouthguards should be specifically recommended, as they provide protection while accommodating ongoing tooth movement and fixed appliances [29]. Clinicians should routinely educate patients regarding the increased risk of dental trauma associated with contact sports and emphasize the importance of immediate dental evaluation following any traumatic impact to the dentition. Such preventive measures may significantly reduce the likelihood of severe luxation injuries and their long-term complications [27,28,29,30].

Additionally, the timing of orthodontic force application following traumatic injury requires careful consideration. Current recommendations suggest that orthodontic movement of traumatized teeth should be delayed until periodontal healing has been sufficiently established and clinical stability is confirmed [15,16,18,21]. Premature reactivation of orthodontic forces may increase the risk of root resorption, compromised periodontal repair, or ankylosis, particularly in teeth that have sustained severe luxation injuries [14,15,16,18,21,22]. In the present case, orthodontic forces were temporarily limited to stabilization purposes, allowing healing to proceed without additional mechanical stress on the injured incisors.

Another important consideration is the patient’s preexisting occlusal relationship. The presence of an edge-to-edge anterior bite and anterior crossbite increased the susceptibility of the maxillary incisors to traumatic impact and post-injury occlusal loading. The strategic placement of posterior bite turbos was therefore a critical adjunctive measure, ensuring functional disocclusion and protecting the traumatized teeth during the initial healing phase. This highlights the importance of individualized occlusal management in trauma cases occurring during orthodontic treatment.

## 5. Clinical Implications

This case provides several clinically relevant insights for orthodontists and general dental practitioners. First, orthodontic appliances should not automatically be removed in the event of dental trauma; instead, they may be strategically incorporated into stabilization protocols. Second, skeletal anchorage systems can offer additional control during the healing phase and may reduce the risk of unintended tooth movement. Third, occlusal management through bite turbos or temporary disocclusion should be considered to protect injured teeth from functional overload.

Interdisciplinary collaboration between orthodontists, endodontists, and general dentists is essential to optimize outcomes in traumatic dental injuries occurring during orthodontic treatment. Clear communication and coordinated treatment planning can significantly influence both short- and long-term prognosis.

## 6. Limitations and Future Considerations

The primary limitation of this case report is the absence of CBCT imaging. Although mobility grades, periodontal probing depths, percussion findings, and radiographic assessments were documented, additional imaging modalities such as CBCT may have provided a more comprehensive evaluation of periodontal and alveolar healing. Nevertheless, the available clinical and radiographic findings demonstrated stable tooth position, absence of pathologic mobility, and favorable healing throughout the follow-up period.

Although favorable clinical and radiographic outcomes were observed during the 16-month follow-up period, longer observation remains necessary to evaluate long-term pulpal and periodontal stability, root resorption, ankylosis, and overall tooth survival.

Additionally, as a single-case report, the findings cannot be generalized to all orthodontic trauma cases. Future studies and case series are needed to better define evidence-based protocols for managing luxation injuries in orthodontic patients, particularly regarding the role of skeletal anchorage and appliance-assisted stabilization.

## 7. Conclusions

This case report demonstrates that combined extrusive and lateral luxation of maxillary incisors during active orthodontic treatment can be successfully managed through immediate multidisciplinary intervention. Prompt repositioning, endodontic therapy, orthodontic stabilization, skeletal anchorage, and occlusal protection resulted in favorable clinical and radiographic outcomes. The orthodontic appliance may have contributed to stabilization of the traumatized teeth and retention within the dental arch; however, this potential protective effect should be interpreted cautiously and requires further investigation. Preventive counseling and routine use of orthodontic-compatible mouthguards should be considered essential components of orthodontic care for patients participating in contact sports.

## Figures and Tables

**Figure 1:**
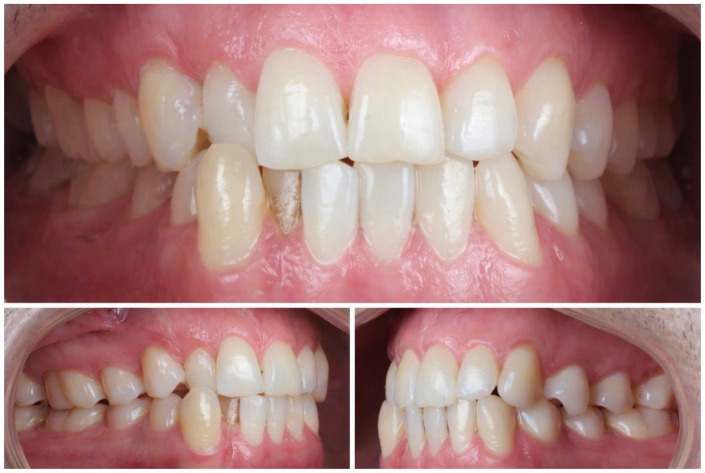
Pretreatment intraoral photographs demonstrated an edge-to-edge anterior relationship, mandibular anterior crowding, anterior crossbite on the right side, asymmetric molar relationships, and absence of the permanent maxillary right canine #6 with significant space loss in that region.

**Figure 2:**
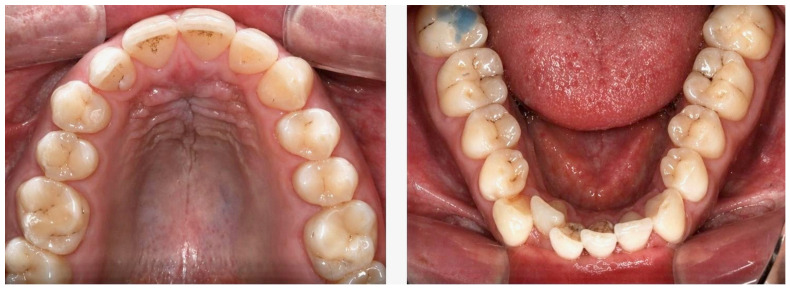
Intraoral occlusal photographs revealed multiple untreated carious lesions, poor oral hygiene, and noticeable attritional wear of the anterior teeth.

**Figure 3:**
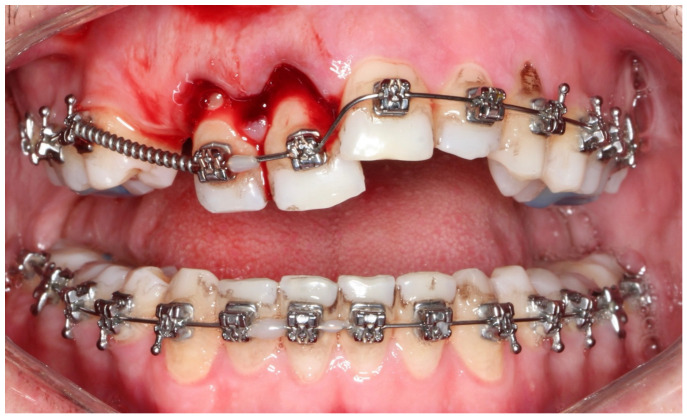
Immediate posttraumatic intraoral frontal view showing combined extrusive and lateral luxation of the maxillary right lateral incisor (#7) and maxillary right central incisor (#8), with marked coronal and lateral displacement, pathologic mobility, gingival laceration, and active bleeding.

**Figure 4:**
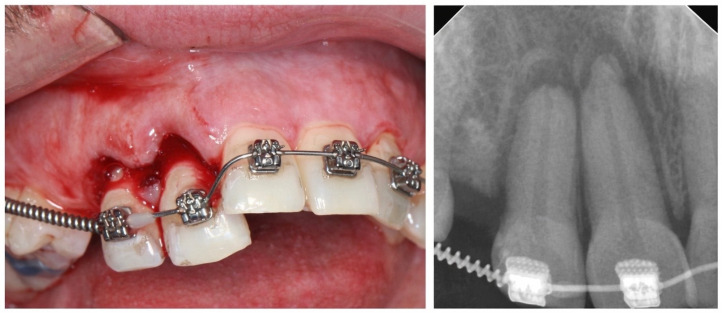
Immediate posttraumatic close-up clinical view and periapical radiograph confirming severe extrusive and lateral displacement of the maxillary right lateral incisor (#7) and maxillary right central incisor (#8).

**Figure 5:**
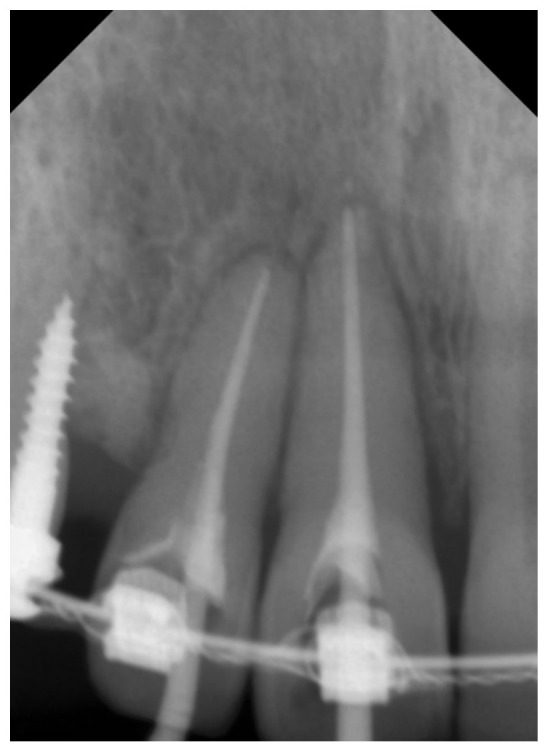
Periapical radiograph after emergency multidisciplinary treatment showing successful repositioning of teeth #7 and #8, completed endodontic therapy, and placement of a mini-screw at site #6 for anchorage.

**Figure 6:**
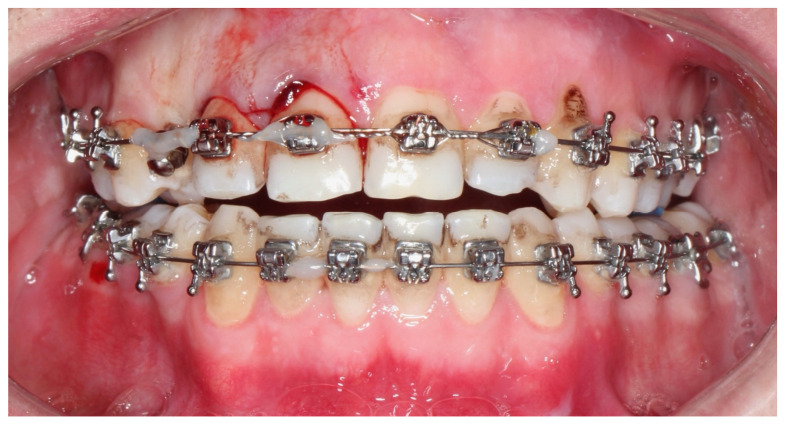
Immediate posttreatment intraoral frontal view showing semi-rigid stabilization of the repositioned maxillary anterior teeth with metallic ligature wires and a 0.017 × 0.025-inch rectangular stainless-steel orthodontic archwire, supplemented by a mini-screw at site #6 to enhance stabilization of the traumatized segment.

**Figure 7:**
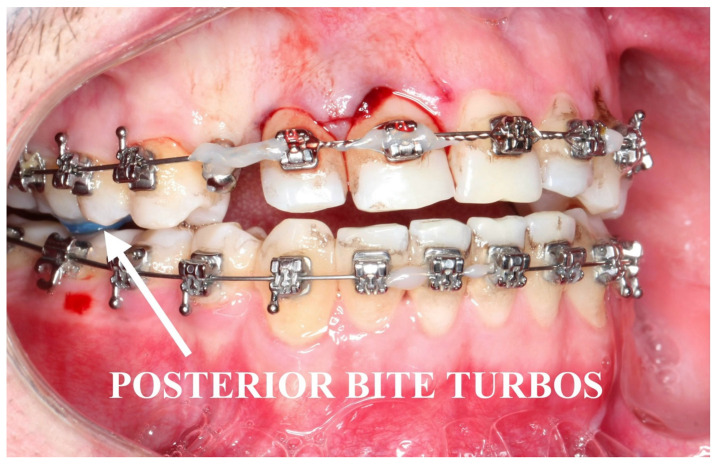
Intraoral lateral views showing posterior bite turbos placed on the maxillary first molars to create temporary posterior disocclusion and minimize functional loading on the injured anterior teeth.

**Figure 8:**
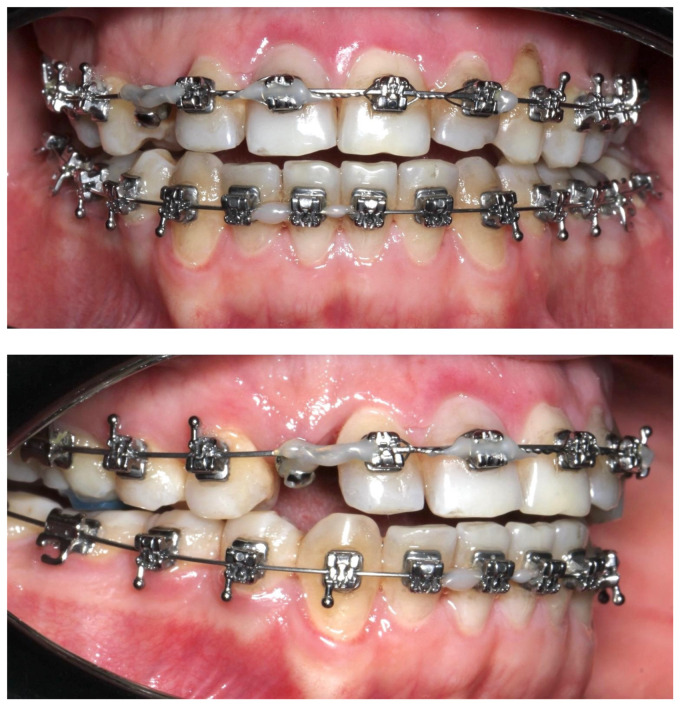
One-month follow-up intraoral views demonstrating stable retention of teeth #7 and #8 within the maxillary arch, maintained semi-rigid splint stabilization, and satisfactory progressive healing of the surrounding soft tissues.

**Figure 9:**
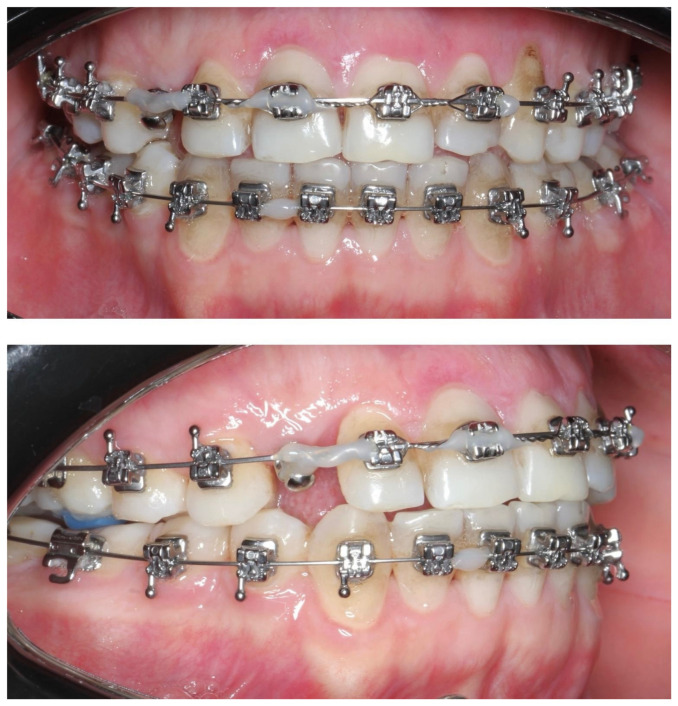
Six-month follow-up intraoral views demonstrating complete clinical stability of teeth #7 and #8, healthy surrounding periodontal tissues, and favorable healing that allowed resumption of orthodontic treatment.

**Figure 10:**
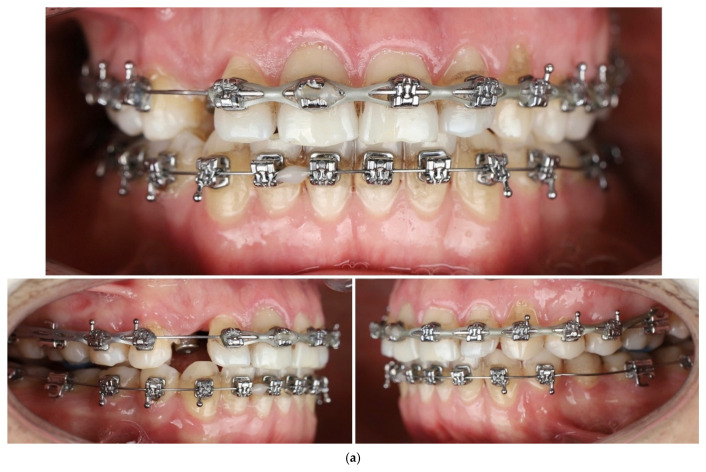

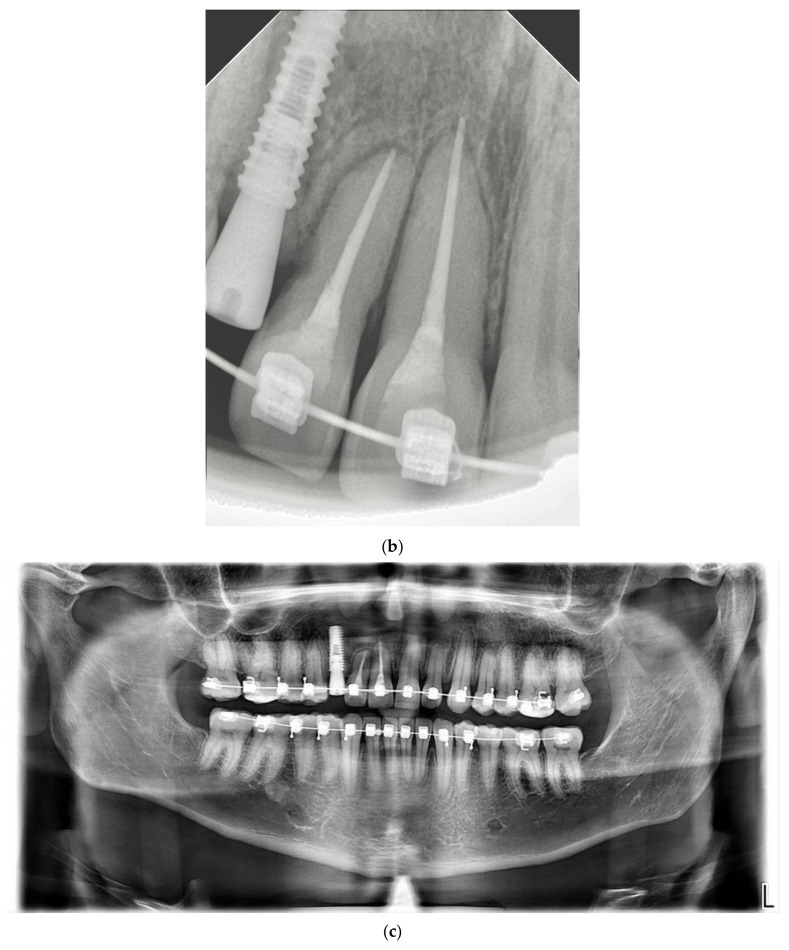
(**a**) Sixteen-month follow-up intraoral views showing stable retention of teeth #7 and #8 during ongoing orthodontic treatment, with maintained ligature stabilization and a dental implant in place at site #6. (**b**) Sixteen-month follow-up periapical radiograph demonstrating stable positioning of teeth #7 and #8, absence of inflammatory root resorption, periapical pathology, or significant periodontal breakdown, and favorable healing of the surrounding supporting structures. (**c**) Sixteen-month follow-up panoramic radiograph confirming stable positioning of teeth #7 and #8, absence of pathologic changes in the maxillary anterior region, and proper placement of the dental implant at site #6.

**Table 1:** Summary of Clinical Outcomes During Follow-Up of Teeth #7 and #8.

Parameter	Baseline	1 Month	6 Months	16 Months
Mobility Grade (#7)	III	I	0	0
Mobility Grade (#8)	III	I	0	0
Periodontal Probing Depth (#7)	≥5 mm	3.5 mm	3 mm	3 mm
Periodontal Probing Depth (#8)	≥5 mm	4.5 mm	3 mm	3 mm
Pulp Status	Vitality could not be assessed d/t acute traumatic injury	Endodontically treated	Endodontically treated	Endodontically treated
Percussion Sensitivity	Positive	Positive	Negative	Negative
Gingival Condition	Laceration, bleeding	Mild erythema	Healthy	Healthy

## Data Availability

The original contributions presented in this study are included in the article. Further inquiries can be directed to the corresponding author.
